# Role of Statin in Reducing Cardiovascular Diseases in Human Immunodeficiency Virus (HIV) Patients: A Systematic Review

**DOI:** 10.7759/cureus.30549

**Published:** 2022-10-21

**Authors:** Ahmed Abdelghafar, Moiud Mohyeldin, Osama S Haroon, Feras O Mohamed, Mahmoud Alfardous Alazm

**Affiliations:** 1 Physiology, University of Medical Sciences and Technology (UMST), Khartoum, SDN; 2 Anatomy, University of Medical Sciences and Technology (UMST), Khartoum, SDN; 3 Radiology, University of Medical Sciences and Technology (UMST), Khartoum, SDN; 4 Pathology, University of Medical Sciences and Technology (UMST), Khartoum, SDN

**Keywords:** hiv patient, cvd, hiv, atherosclerosis, coronary intima media thickness, statin, cardiovascular diseases

## Abstract

Because of abnormal lipid profiles, the risk of patients living with human immunodeficiency virus (PLHIV) developing cardiovascular diseases (CVDs) is much greater than in the general population. Statins are known effective medications for reducing the risk of CVDs by influencing inflammatory mediators, endothelial function, angiogenesis, and thrombosis. However, their role in PLHIV has not been well confirmed. In this article, we aim to provide more clarity about the effects of statin in lowering CVDs in PLHIV. Cochrane Library, PubMed, PubMed Central (PMC), and Medical Literature Analysis and Retrieval System Online (MEDLINE) were screened and searched for articles that contained relevant data to our study. In this article, three clinical trials and five observational studies, including eight abstracts, were obtained and analyzed. We included articles that examined the efficacy of statins in reducing CVDs in those with PLHIV. Two reviewers independently abstracted and collected the data. We infer that the cardiovascular events in PLHIV were reduced by the potent effects of statins.

## Introduction and background

The World Health Organization (WHO) states that 38 million people are infected with the human immunodeficiency virus (HIV) [1]. Cardiovascular diseases (CVDs), such as elevated serum total cholesterol (TC) levels and coronary artery disease (CAD), are the leading causes of developing CVD, which is the primary cause of high death rates in developed countries [2]. Disabilities resulting from CVD are high and are the primary reason for the loss of productivity [3].

Scientists state that the main reason HIV patients develop CVD is abnormal lipid profiles, including elevated levels of low-density lipoprotein (LDL) cholesterol, triglycerides (TGs), and TC levels [4]. This makes HIV patients more prone to develop CVD. An abnormal lipid profile is associated with an increased hazard of developing CVD, which is a significant cause of mortality among the general population [4]. A consistent body of evidence explains that patients living with human immunodeficiency virus (PLHIV) are 50%-100% more likely to have increased incidence of CVD than people without HIV infection, even when other risk factors are controlled for, such as elevated levels of cholesterol, hypertension, and smoking status [5].

Lipoprotein-associated phospholipase A2 (Lp-PLA2) is an inflammatory marker used to predict the development of cardiovascular events in the general population [6]. Statins are a group of lipid-lowering drugs causing effective suppression in the efficiency of 3-hydroxy-3-methylglutaryl co-enzyme A (HMG-COA) reductase, the precursor for the synthesis of cholesterol, and inhibit the output of cholesterol, resulting in reduced serum cholesterol levels [7]. Statins are potent therapies for reducing the risk of mortality and future CVD in those with known coronary heart disease [8]. This effect is achieved by lowering cholesterol levels, but it can also be achieved by the pleiotropic effects of lipid-lowering agents, lowering chronic inflammation, and oxidative stress [9-10]. Low‐density lipoprotein cholesterol (LDL‐C) is suppressed by the clinical effect of statin therapy; however, many articles have described pleiotropic impacts, separate from the action of statin in reducing LDL-C [11]. Statins are known to influence inflammatory mediators [12], endothelial function [13], angiogenesis [14], and thrombosis [15]. However, whether statin therapy is associated with CVD reduction among HIV patients is unclear, and we need more trials to confirm this effect.

The ongoing Randomized Trial to Prevent Vascular Events in HIV (REPRIEVE) will assess the efficacy of statins in the reduction of CVD in PLHIV [16]. The efficacy of statins to minimize atherosclerosis and promote cardiac events in PLHIV has not been well established [17]; therefore, we performed a systemic review to offer an overview of the effectiveness of statins in reducing CVD in HIV patients.

## Review

Method

Protocol

In this systematic review, we used the Preferred Reporting Items for Systematic Reviews and Meta-Analysis (PRISMA) guideline to establish the quality of this review [18].

Inclusion and Exclusion Criteria

Inclusion criteria centered on adult population studies, published in English as full-text papers, from the past 10 years. We included randomized controlled clinical trials, cohort, and cross-sectional studies. However, studies on animal species, the pediatric population or those written in other languages were excluded.

Data Origin and Strategies

In this article, we screened the subsequent four databases, Cochrane Library, Medical Literature Analysis and Retrieval System Online (MEDLINE), PubMed, PubMed Central (PMC) and Google scholar, to search for relevant data for our article. Research in the database PubMed was conducted on the seventh of July, 2021. The search for relevant articles was done by using medical subject headings (Mesh) to search for relative concepts ("HIV patient," "Cardiovascular diseases", '' Atherosclerosis'' and "Statin"). Depending on titles, abstracts and full-text features, articles were fully screened to rule in/out relevant information. We extracted the items from each article containing study design, year of publication, intervention methods, and research findings. Studies collected by two reviewers were analyzed by other reviewers for worthiness.

Bias Assessment Tools

The quality check was done by using the subsequent tools shown in Table 1.

**Table 1:** Quality appraisal tools. The following tools were used to article check for each respective paper type: JBI checklist (n=4) for all four articles; a 30% risk of bias was the maximum tolerated percentage. The COCHRANE risk bias assessment tool (n=4) was used in all four studies, for which seven criteria were chosen to rule out potential biases. All eight articles were included in a systemic review and satisfied the cut-off (>70%). JBI, Joanna Briggs Institute

Quality appraisal tools	Articles
Cochrane risk bias assessment tool	Randomized controlled trials
JBI checklist	Cohort and cross-sectional studies

Results

**Figure 1:**
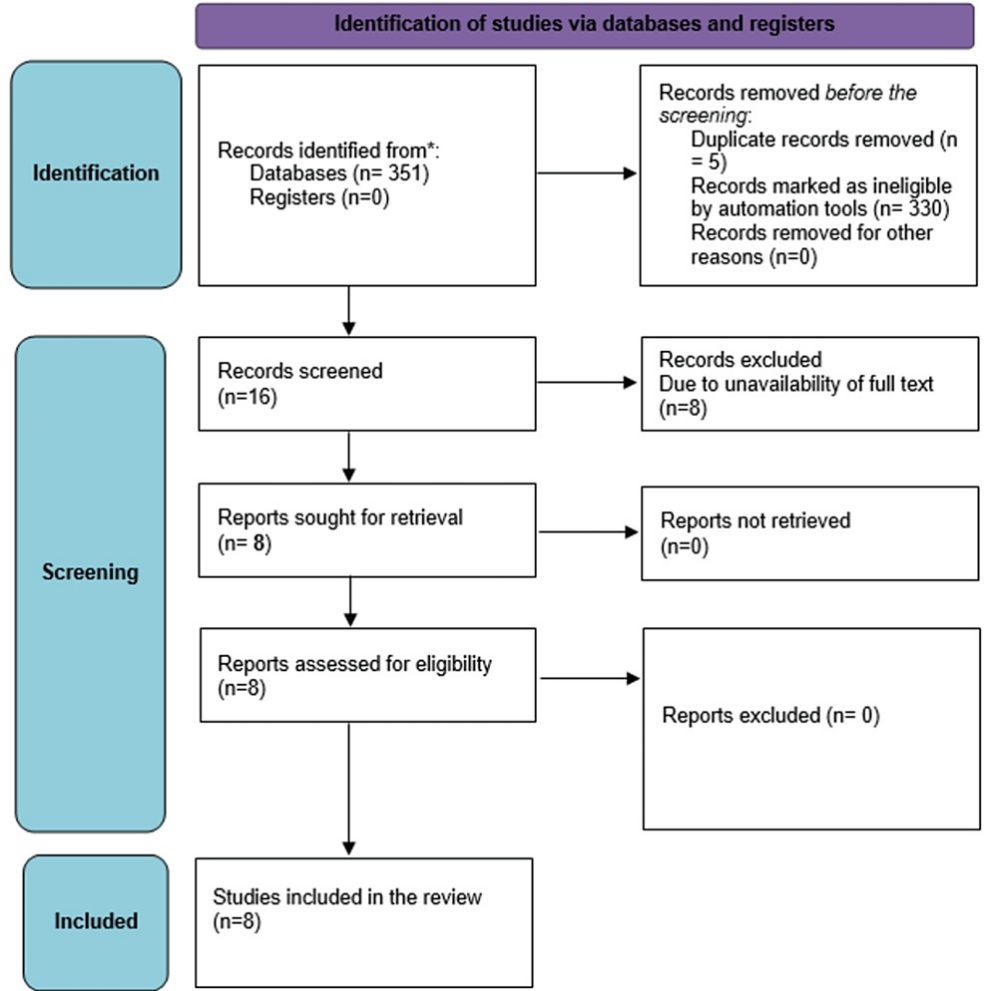
The PRISMA diagram. PRISMA, Preferred Reporting Items for Systematic Reviews and Meta-Analysis

Using different databases to search for relevant data, we identified 351 articles. After removing five duplicate articles, 346 articles were left, from which 211 articles were from PubMed, 39 studies from MEDLINE, 81 studies from Cochrane, and 15 studies from Google Scholar. We screened the remaining articles depending on the title's relevance and the contents of their corresponding abstracts to our research. Some 330 articles were discarded due to irrelevance. Hence, 16 articles were left, and we checked for the availability of full articles, out of which eight articles were removed due to the unavailability of full articles. Eight articles were found appropriate, based on the eligibility criteria. The Preferred Reporting Items for Systematic Reviews and Meta-Analysis (PRISMA) flow chart is shown below in Figure 1.

Characteristics of Included Studies

Demographic features are shown below in Table 2.

**Table 2:** Demographic features. HAART, highly active antiretroviral therapy

Demographic features / author names; publication year	Male	Female	Hypertension	Diabetes mellitus	HAART	Statin (-)	Statin (+)
Phan et al. (2020) [17]	119	5	61	20	-	99	28
Calza et al. (2012) [19]	91	44	15	-	89	93	42
Foldyna et al. (2020) [20]	32	8	6	4	-	21	19
Calza et al. (2021) [21]	101	24	60	-	125	0	125
Lo et al. (2016) [22]	32	8	6	4	40	21	19
Ou et al. (2016) [23]	844	101	224	154	-	0	945
Krsak et al. (2015) [24]	297	141	151	30	438	371	67
Eckard et al. (2014) [25]	115	32	-	-	-	75	72

The authors found that hypertension in this study was associated with a faster carotid intima media thickness (IMT progression) and was a significant factor in mortality among HIV patients. However; statin users have a reduction in all-cause of mortality, including hypertension [17]. Calza and his co-authors reported neither serious toxicity nor serious adverse events related to statin therapy that necessitates discontinuance of statin therapy [19]. The researchers found that PLHIV who received stable combined antiretroviral therapy (cART) and rosuvastatin for 12 months had a reduction in atherosclerosis progression. Therefore, we conclude that a combination between cART and rosuvastatin has a safe effect and positive outcome among PLHIV [21]. Krsak and his colleagues reported drug-drug interaction among PLHIV who received statin therapy and cART, mainly protease inhibitors (PIs) [24].

We tabulated the summary of eight studies below in Table 3.

**Table 3:** Summary of clinical studies in PLHIV. IMT, intima-media thickness; cIMT, coronary intima-media thickness; CVD, cardiovascular disease; Lp-PLA 2, lipoprotein-associated phospholipase A2; LDL, low-density lipoprotein; HIV, human immunodeficiency virus; MI, myocardial infarction; PLHIV, patient living with human immunodeficiency virus

Author names; Publication year	Study design	Intervention	Primary findings
Phan et al. (2020) [17]	Cohort study	Statin, followed-up for 3.2 years	cIMT progression in the statin and non-statin groups was similar, with no significant progression in cIMT (0.062 mm/yr vs 0.058 mm/yr)
Calza et al. (2012) [19]	Cross-sectional study	Rosuvastatin 10 mg daily, total population (n=42),then followed up for 24 months	Significant reduction in a mean IMT of carotid bifurcations, internal carotid, common carotid arteries and carotid plaque were observed
Foldyna et al. (2020) [20]	Randomized placebo-controlled trial	Atorvastatin 40 mg daily (n=19) vs placebo (n=21), serial coronary CT angiography at baseline and after one year was done in both statin and placebo groups	In the same HIV patients, some plaques increased while other plaques decreased. Overall, 77% vs 51% of individual plaques increased, while 24% vs 49% decreased in placebo and statin, respectively
Calza et al. (2021) [21]	Cohort study	Rosuvastatin 10 mg daily, total population (n=125); metabolic syndrome group (n=61); control group (n=64). Followed up for 12 months	In the metabolic syndrome group, mean IMT elevated at month 12 compared with respective baseline values, which is not significant in all evaluated anatomical sites ( carotid bifurcation, common carotid, and internal carotid arteries). However, the average IMT was statistically considerable in the control group in all evaluated anatomical sites (common carotid, internal carotid arteries, and carotid bifurcation)
Lo et al. (2016) [22]	Randomized, double-blind, placebo-controlled clinical trial	Atorvastatin 20 mg was initially received by HIV patients and then titrated to 40 mg after four months. Total population (n=40); atorvastatin (n=19); placebo (n=21)	There were no changes in primary outcomes, but noncalcified plaque volume was reduced eventually
Ou et al. (2016) [23]	Cohort study	Intensive statin regimens on CVD risks, total population (n=945)	The researchers found that the CVD risk was lower in the HIV patients who received a high statin dose than PLHIV who received a low statin dose.
Krsak et al. (2015) [24]	Cohort study	Statin	They found no considerable correlation between lipid-lowering agents and the complex results of stroke and MI
Eckard et al. (2014) [25]	Randomized, double-blinded, placebo-controlled trial	Rosuvastatin 10 mg once a day orally (n=72), placebo group (n=75). They followed up for 24 weeks	The Lp-PLA2 level was reduced in the statin group, and in the placebo group, there was a reduction of 2% in the Lp-PLA2 level. In the HIV patients group, LDL cholesterol levels were reduced compared to an elevation in the placebo group

Discussion

To treat dyslipidemia, we used statins for minimizing CVD risk in PLHIV. The leading cause for an elevated death rate and morbidity in HIV patients is CVD [26]. Therefore, the promising effect of statins in reducing CVD among HIV patients is the main concern of this article.

Role of Statins in Reducing Coronary Intima-Media Thickness (cIMT)

Rosuvastatin is a very efficient drug in lowering C-reactive proteins, and LDL cholesterol levels in the plasma and decelerates the progression of cIMT in the general population. Atherosclerosis in carotid arteries is determined when cIMT is more than or equal to 0.9 mm thickness [19].

Calza et al. conducted a cross-sectional study to assess the impact of statins on atherosclerosis progression in HIV patients with asymptomatic atherosclerosis in the carotid arteries and high cholesterol levels [19]. After fulfilling the inclusion criteria, the participants initially received rosuvastatin treatment at 10 mg once a day and then followed up for 24 months. Ultrasound assessment of the carotid arteries was done on all participants to define the baseline measurement and then repeated within two months after the period of follow-up. Rosuvastatin led to a considerable lipid plaque reduction in the carotid artery bifurcations, and internal carotid arteries and played a significant role in improving the lipid parameters in PLHIV [19]. However, this study is limited to patients with moderate cardiovascular risk, so the efficiency of rosuvastatin in patients who have a low cardiovascular risk cannot be determined. Therefore, while rosuvastatin plays a significant role in reducing carotid atherosclerosis in HIV patients, further randomized trials testing the efficiency of statins in minimizing atherosclerotic progression in HIV patients must be conducted.

In a similar randomized controlled trial (RCT), conducted by Foldyna et al., on PLHIV with asymptomatic coronary arterial atherosclerosis, the participants received atorvastatin therapy (20 mg orally once a day in the first three months and increased to 40 mg orally once a day for the final nine months, if tolerated) or a placebo [20]. All participants were subjected to serial coronary measurements at baseline and one year after fulfilling the inclusion criteria. The researchers measured the plaque volume and structure to estimate the variations of individual coronary plaques in PLHIV there was a 24% vs. 49% regressed in placebo and statin, respectively [20]. Therefore, fatty and fibrotic components' progressions were stabilized by the action of atorvastatin, with no effect on observed calcifications [20]. The researchers concluded that in PLHIV, the coronary plaque progression and regression varied within particular patients.

Calza et al. conducted a prospective cohort study to examine the efficiency of statins in PLHIV with or without metabolic syndrome (control group), for which PLHIV received rosuvastatin 10 mg orally once a day; then followed up for 12 months [21]. In all the evaluated anatomical sites (carotid bifurcations, common carotid arteries, and internal carotid arteries), the average increase in cIMT was considerably lower in the metabolic syndrome group than in the control group. This study was limited to one statin at one dosage, so another study with different statins at several doses would be indispensable [21]. Nevertheless, this is the first study investigating the effect of statins on soluble inflammation markers and cIMT progression rates among HIV patients with metabolic syndrome. This study suggests a potential action of rosuvastatin in reducing vascular atherosclerosis in PLHIV.

Efficacy

Statins have a considerable effect in lowering CVD and death rates in the general population [8]. However, the exact role of statins in HIV patients is still ambiguous, and not well explained. In this part, we discuss some of the observational and clinical studies in this field.

Lo et al. conducted a RCT that involved 40 PLHIV who received cART [22]. They were then randomly selected to receive 20 mg of atorvastatin, titrated to 40 mg after three months, or to a placebo group. The patients were followed for 12 months to examine the effect of atorvastatin in reducing CVD in HIV patients [22]. There was no difference between the primary cardiovascular findings of the aortic target and the background ratio of fluorodeoxyglucose uptake measured by a positron emission tomography (PET) scan or CT scan; however, there was a spectacular depression in noncalcified plaque volume measured by coronary CT angiography and in the reduced number of plaques with high-risk features [22]. Noncalcified plaque is more vulnerable to rupture. Thus, the statins decrease noncalcified plaque in HIV patients, representing a protective role against CVD in this population [22]. This study showed that statins played an effective role in decreasing CVD for PLHIV who are at low risk; however, this trial was limited by being confined to surrogate markers of CVD.

To assess the efficiency of intensive statin treatment (i.e., high-dose or potency of statins) in reducing cardiovascular events in PLHIV, a cohort study was conducted by Ou et al. on 945 patients [23]. The scientists found in PLHIV with CVD history, the group who received a high dosage of statin therapy had a lower CVD risk compared to patients who received low statin dosage. The group who received a high-potency (i.e., atorvastatin) statin drug revealed a reduction in CVD risk compared to a low-dosage [23]. Therefore, high-dosage or high-potency statin therapy plays a considerable role in primary and secondary protection against cardiovascular events in PLHIV [23]. The study defines hypertension as a risk factor for CVD. However, other risk factors, such as occupation, smoking, physical activity behaviors, and family history for CVD were not considered, which might lead to a specific degree of residual confounding.

A retrospective cohort study was designed to assess any relationship between statin use and myocardial infarction and stroke [24]. The researchers found no relationship between lipid-lowering agent use and any reduction in stroke, myocardial infarction and all causes of death in PLHIV [24]. Nevertheless, the role of statins should be examined by experimental trials, such as the REPRIEVE trial.

Eckard et al. conducted a randomized, double-blinded, placebo-controlled trial [25]. The main focus of this article was to assess the improvement in Lp-PLA2 enzymes, activation of T-cells, inflammation of both the systemic and vascular systems, and LDL-cholesterol levels in HIV patients who received rosuvastatin (10 mg once a day vs. placebo group) [25]. A significant decrease in the levels of Lp-PLA2 was reported, which is a biomarker of vascular dysfunction that predicts cardiovascular incidents in the general population [25]. This study investigated HIV patients who received antiretroviral therapy (ART) with low or undetectable levels of HIV-1 RNA and normal plasma levels of LDL cholesterol. This study revealed the anti-inflammatory role of statins, which contributes to reducing the progression of CVD in HIV patients.

Phan et al. conducted a longitudinal, observational cohort study on 127 PLHIV with a high risk of developing CVD, according to atherosclerotic cardiovascular disease (ASCVD) risks [17]. The study was conducted to estimate whether or not statins were related to a reduction in cIMT progression in PLHIV. The mean progression of cIMT was equal in both the statin group and the non-statin group [17]. Future research must be performed to demonstrate the efficiency of statins on cardiovascular events in PLHIV [17]. This research study was limited to males and Caucasians; therefore, females and non-Caucasians were not assessed. In addition, there were a few deaths and not all of the deaths were due to cardiovascular events.

The need for preventing CVD in PLHIV is crucial. Further studies are needed to specifically estimate the efficiency of statins in preventing cardiovascular events in PLHIV [27]. In conclusion, statins appear to have an efficient effect in lowering cardiovascular events in PLHIV. Future trials are needed to support this finding.

Study Limitations

Our study had several limitations. We eliminated all papers focused on the pediatric population, unpublished literature, and gray literature. The studies included in this paper involved only studies written in the English language from 2011 to 2021. In addition, our search was limited and we did not use any mesh terms.

## Conclusions

Human immunodeficiency virus patients have a higher incidence of developing cardiovascular events than the general population due to lipid abnormalities, including elevated LDL, TGs levels, and Lp-PLA2 enzymes. Statins inhibit cholesterol production, down-regulate serum cholesterol, and decrease Lp-PLA2. Therefore, we conducted this review to clarify the pivotal role of statin in lowering cardiovascular events in PLHIV. We found that statins have a beneficial role in reducing atherosclerosis, stabilizing the progression of plaques, decreasing the level of Lp-PLA2, and reducing cIMT in HIV patients. However, further trials and long-term observational studies should be undertaken to further assess the benefits of statins in reducing cardiovascular events in HIV patients.
